# Health Tract No. 314

**Published:** 1868-01

**Authors:** 


HEALTH TRACT No. 314.

### MARRIAGE MAXIMS.

A good wife is the greatest earthly blessing. A man is what his wife makes him. It is the mother who moulds the character and destiny of the child.

Make marriage a matter of moral judgment.

Marry in your own religion.

Marry into a different blood & temperament from your own.

Marry into a family which you have long known.

Never talk at one another, either alone, or in company.

Never both manifest anger at once.

Never speak loud to one another, unless the house is on fire.

Never reflect on a past action, which was done with a good motive and with the best judgment at the time.

Let each one strive to yield oftenest to the wishes of the other.

Let self-abnegation be the daily aim and effort of each.

The very nearest approach to domestic felicity on earth is in the mutual cultivation of an absolute unselfishness.

Never find fault unless it is perfectly certain that a fault has been committed; and even then, prelude it with a kiss, and lovingly.

Never taunt with a past mistake.

Neglect the whole world beside, rather than one another.

Never allow a request to be repeated.

"I forgot" is never an acceptable excuse.

Never make a remark at the expense of the other; it is a meanness.

Never part for a day without loving words to think of during absence; besides, you may not meet again in life.

They who marry for physical characteristics, will fail of happiness; they who marry for traits of mind and heart, will never fail of perennial springs of domestic enjoyment.

They are safest who marry from the standpoint of sentiment rather than from that of feeling, passion or mere love.

The beautiful in heart is a million times of more avail in securing domestic enjoyment, than the beautiful in person or manners.

Do not herald the sacrifices you make to each others tastes, habits or preferences.

Let all your mutual accomodations be spontaneous, wholesouled, and free as air.

A hesitating, tardy or grum yielding to the wishes of the other always grates upon a loving heart, like Milton's "Gates on rusty hinges turning."

Whether present or absent, alone or in company, speak up for one another, cordially, earnestly, lovingly.

If one is angry, let the other part the lips, only to give a kiss.

Never deceive, for the heart once misled, can never wholly trust again.

Consult one another in all that comes within the experience and observation and sphere of the other.

Give your warmest sympathies for each other's trials.

Never question the integrity, truthfulness, or religiousness of one another.

Encourage one another in all the depressing circumstances under which you may be placed.

By all that can actuate a good citizen, by all that can melt the heart of pity, by all that can move a parent's bosom, by every claim of a common humanity, see to it, that at least one party shall possess strong, robust, vigorous health of body and brain ; else let it be a marriage of spirit with spirit; that only, and no farther.

